# Routine HIV testing in the context of syndromic management of sexually transmitted infections: outcomes of the first phase of a training programme in Botswana

**DOI:** 10.1136/sti.2007.028217

**Published:** 2008-03-20

**Authors:** M R Weaver, M Myaya, K Disasi, M Regoeng, H N Matumo, M Madisa, N Puttkammer, F Speilberg, P H Kilmarx, J M Marrazzo

**Affiliations:** 1 University of Washington, Seattle, Washington, USA; 2 International Training and Education Center on HIV (I-TECH), Seattle, Washington, USA; 3 BOTUSA (United States Centers for Disease Control and Prevention (CDC)/Botswana), Gaborone, Botswana; 4 Ministry of Health of the Republic of Botswana, Gaborone, Botswana; 5 National Center for HIV/AIDS, Viral Hepatitis, STD, and TB Prevention, CDC, Atlanta, Georgia, USA; 6 University of California at San Francisco, San Francisco, California, USA

## Abstract

**Objective:**

In 2004, the Ministry of Health adopted revised protocols for the syndromic management of sexually transmitted infections (STI) that included routine HIV testing. A training programme for providers was developed on the revised protocols that featured interactive case studies and training videos. An objective of the first phase of the training programme was to test its effect on four measures of clinical practice: (1) routine HIV testing; (2) performance of physical examination; (3) risk-reduction counselling and (4) patient education.

**Methods:**

Clinical practice in a district where providers were trained was compared with a district without training. The measures of clinical practice were reported by 185 patients of providers who had been trained and compared with reports by 124 patients at comparison clinics.

**Results:**

Relative to patients at comparison clinics, a higher percentage of patients of trainees reported that the provider: (1) offered an HIV test (87% versus 29%; p<0.001); (2) conducted a physical examination (98% versus 64%; p<0.001); (3) helped them to make a plan to avoid future STI acquisition (95% versus 76%; p<0.001) and (4) provided patient-specific information about HIV risk (65% versus 32%; p<0.001). Among patients offered HIV testing, the percentage who accepted did not differ between groups (38% of 161 patients of trainees versus 50% of 36 comparison patients; p = 0.260). Overall, 33% of patients of trainees and 14% of comparison patients were tested (p<0.001).

**Conclusion:**

A multifaceted training programme was associated with higher rates of HIV testing, physical examination, risk-reduction counselling and better HIV risk education.

Several African countries have adopted syndromic management for sexually transmitted infections (STI) as part of their comprehensive reproductive health, STI and HIV prevention strategies.^1^^–^7 Syndromic management is based on a presumptive diagnosis of STI and is typically directed by national protocols for treatment based on symptoms and easily recognised signs of infection.^8^ Regardless of whether the diagnosis is presumptive or aetiological, correct case management of STI includes nine elements: history; physical examination; diagnosis; early and effective treatment; advice on sexual behaviour; promotion and provision of condoms; partner notification and treatment; case reporting and, if necessary, clinical follow-up.^8^

In Botswana, the Ministry of Health (MOH) reviews and updates its national syndromic management protocols based on periodic aetiological studies and other developments in healthcare.^9^^–^11 A study performed in 2002 that used highly sensitive diagnostic assays demonstrated a high prevalence of HIV among patients seeking care for STI-related complaints relative to a sentinel HIV prevalence of 38.6% among pregnant women in Botswana in 2001.^12^ HIV prevalence was 54% among women with vaginal discharge or lower abdominal pain (VD/LAP), 62% among men with urethral discharge and 74% among patients with genital ulcer disease (GUD).^11^ The proportion of GUD cases caused by genital herpes increased between 1993 and 2001, whereas the proportion caused by syphilis and chancroid decreased.^11^

In 2004, the MOH adopted revised protocols that included routine HIV testing as part of every STI consultation. Botswana began routine, non-compulsory HIV testing (ie, “opt-out”) as part of all medical services in January 2004^13^ ^14^ and revised protocols presented an opportunity to integrate STI and HIV care further. In the revised GUD protocol, patients with GUD receive episodic treatment for genital herpes with acyclovir (400 mg by mouth three times a day for seven days) as well as treatment for syphilis and chancroid; patients whose GUD is characterised by vesicles receive only episodic treatment with acyclovir.

To implement the revised protocols, the MOH developed a new national STI training programme in cooperation with the BOTUSA (United States Centers for Disease Control and Prevention (CDC)/Botswana) and the International Training and Education Center on HIV/AIDS (I-TECH). An objective of the first phase of the training programme was to test its effect on four measures of clinical practice: (1) routine HIV testing; (2) performance of physical examination; (3) risk-reduction counselling and (4) patient education.

## METHODS

### Study design

Clinical practice in a district where providers were trained was compared with a district without training. Several weeks after training, its effects on clinical practice were measured by patient reports during exit interviews after visits that included STI care. A trained interviewer read standardised questions to the patient about whether or not tasks were performed by providers. The interviewer also read statements about the quality of care and asked the patient to rate the care on a five-point scale, in which “strongly agree” was rated 5 and “strongly disagree” was rated 1.

### Selection of districts

The selection of the training (Lobatse Town Council) and comparison (Southeast) districts was based on four criteria: (1) average or higher than average proportion of GUD cases among STI visits; (2) low number of clinics; (3) clinics located relatively close to one another within a district and (4) proximity of districts. The number of clinics was a criterion because providers were trained by district; only approximately 200 people could be trained in the first phase and the low number of clinics corresponded to the low number of people to train per district. Twenty-three per cent of STI visits were for GUD in Lobatse and 17% in Southeast compared with a national average of 17%. Lobatse had nine public clinics and Southeast had 11 compared with a national average of 26. Clinics were located relatively close to one another in both districts. Lobatse and Southeast had a higher-than-average population density (703 and 34 people per square kilometre compared with a national average of three).^15^ Lobatse and Southeast are geographically adjacent, which may have reduced differences among clinics and in health-seeking behaviour between the two patient populations.

### Selection of clinics

Clinics were selected on the basis of two criteria: (1) an average of 10 or more STI cases per month and (2) clinics at which patients could give informed consent. Patients were interviewed at five of the nine facilities in Lobatse, including outpatient clinics of the district hospital and four public sector primary care clinics. Four facilities were excluded: a mental health hospital, a prison clinic and two clinics that reported fewer than 10 STI cases per month. In Southeast district, patients were interviewed at the hospital of a faith-based organisation and six of 11 public health facilities, including a primary care clinic and five health posts, which are the smallest unit of Botswana’s primary care system. Five facilities that reported fewer than 10 STI cases per month were excluded.

### Intervention

All nurses, nurse midwives and medical officers in Lobatse Town Council were entitled to training and 194 out of 212 providers (91.5%) were trained during 11 three-day sessions from 6 September to 28 October 2004. The curriculum was designed for clinical training in resource-limited settings, including interactive case studies and films on patient-centered care, sensitive female pelvic examination, risk-reduction counselling and HIV post-test counselling (available at http://www.go2itech.org).^16^^–^19 Sessions were facilitated by master trainers from the National STI Training and Research Center and core trainers, who were trained as trainers in the new curriculum.

### Recruitment of patients

Female patients 16–49 years old presenting with GUD and/or VD/LAP and male patients 16–49 years old presenting with GUD and/or urethral discharge were eligible. Providers referred all patients who met the inclusion criteria to an interviewer. Participants provided informed consent by signing, initialing or marking a consent form that was countersigned by the interviewer. An exception was when a clinical specialist was at clinics in Lobatse to observe visits (see “Statistical analysis”) and countersigned the consent form.

A total of 216 patients in Lobatse and 128 patients in Southeast district were invited to participate; response rates were 86% and 97%, respectively (185 patients in Lobatse and 124 patients in Southeast district).

### Human subjects review

All procedures and questionnaires were reviewed and approved by the Botswana MOH Health Research Unit. They were also reviewed by the Associate Director for Science, National Center for HIV, STD and TB Prevention, CDC, who determined that the activity was not research.

### Statistical analysis

Bivariate analyses were conducted using SPSS-PC version 13.0 for Windows (SPSS Inc, Chicago, Illinois, USA). All p values were two-sided and a level of less than 0.05 was considered significant. The relative risk was calculated as the ratio of training to comparison clinic percentages.

When there were differences between training and comparison samples, multivariate analyses were performed with a modified Poisson regression approach^20^ ^21^ using SAS software version 9.1 (SAS Institute Inc, Cary, North Carolina, USA) and the adjusted relative risk is reported. The visits of approximately half the patients at training clinics were observed by a clinical specialist. An observer could have improved the providers’ practice during those visits, thus the multivariate analysis included the presence of the observer as a variable.

The dependent variables for the 11 statements about quality of care were analyzed two ways: (1) as a dichotomous choice between agreement or disagreement with the statement or (2) as a continuous response on a five-point scale. The results of the analyses were similar and the former is reported.

To facilitate interpretation, STI syndrome variables were hierarchical; for example a patient who reported both GUD and VD/LAP was only counted as GUD. Only 23 out of 194 women (14%) with VD/LAP also reported GUD. Only eight out of 36 men (18%) with urethral discharge also reported GUD.

## RESULTS

### Patient and provider characteristics

The characteristics of patients who were interviewed and their visits are presented in table 1. Significantly more patients at training clinics were treated by nurse midwives (31%) than at comparison clinics (16%) (p = 0.003) and correspondingly fewer by nurses. Significantly more patients had visits for follow-up care at training clinics than comparison clinics (29% versus 10%, respectively, p<0.001) and correspondingly fewer were first visits. The syndromes that patients reported were similar, with one exception. At training clinics, 8% of the patients reported urethral discharge compared with 18% at comparison clinics (p = 0.006) or 27% compared with 51% of the male patients, respectively.

**Table 1:** Description of patients and their visits at clinics with the STI syndromic management training programme and comparison clinics, Botswana 2004

	Patients from clinics with trained health workers	Patients from comparison clinics	p Value
	n (%)	n (%)	
Characteristics of patients			
Gender of patient (% female)	134 (74)	81 (65)	0.119
Average age in years	28	29	0.286
Language spoken at home			
Setswana	173 (94)	111 (90)	0.207
Kalanga	2 (1)	6 (5)	0.064
English	5 (3)	2 (2)	0.706
Other	5 (3)	5 (4)	0.530
Education (one missing from each district)			
No formal, non-formal and primary	50 (27)	38 (31)	0.490
Junior secondary	78 (42)	54 (44)	0.809
Senior secondary and post-secondary	56 (30)	31 (25)	0.313
Married or cohabiting	54 (29)	41 (33)	0.469
Characteristics of patients’ visits			
Gender of provider who treated patient (% female)	153 (83)	94 (76)	0.138
Profession of provider who treated patient			
Nurse	122 (66)	101 (82)	0.003
Nurse midwife	57 (31)	20 (16)	0.003
Medical officer	6 (3)	3 (2)	0.745
Visit type			
First visit	132 (71)	111 (90)	0.001
Follow-up visit	53 (29)	13 (10)	<0.001
Syndromes			
Genital ulcer	45 (24)	28 (23)	0.724
VD/LAP	109 (59)	62 (50)	0.122
Urethral discharge	14 (8)	22 (18)	0.006
No symptoms	17 (9)	12 (10)	0.885
Visit observed by clinical specialist	98 (53)	0 (0)	<0.001
Referred by contact slip	14 (8)	6 (5)	0.480
Sample size	185	124	

STI, Sexually transmitted infection; VD/LAP, vaginal discharge or lower abdominal pain.

### Routine HIV testing

**Figure 1:**
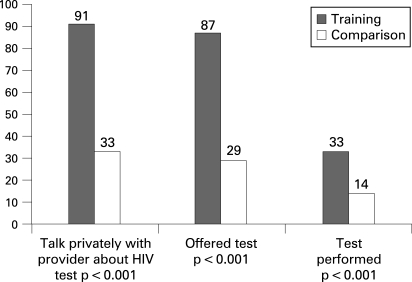
Patient reports on HIV testing at clinics with a sexually transmitted infection syndromic management training programme and comparision clinics, Botswana 2004.

Significantly more patients at training clinics had an HIV test (33%) than at comparison clinics (14%) (p<0.001). As shown in fig 1, 91% of the patients at training clinics reported having enough opportunity to talk privately with the provider about HIV testing compared with 33% at comparison clinics (p<0.001) and 87% of the patients at training clinics were offered an HIV test compared with 29% at comparison clinics (p<0.001). Among patients offered an HIV test, 38% of 161 at training and 50% of 36 at comparison clinics (p = 0.260) agreed to be tested.

A multivariate analysis adjusted for the differences between samples in provider profession, visit type, patient gender, syndromes, observation by a clinical specialist and before coming to the clinic whether or not the patient understood that s/he had the right to refuse an HIV test. As shown in columns 2 and 3 of table 2, patients at training clinics were 2.55 (95% confidence interval (CI) 1.96 to 3.32) times more likely than at comparison clinics to talk privately with a provider about HIV testing. As shown in columns 4 and 5, patients at training clinics were 2.92 (95% CI 2.19 to 3.90) times more likely than at comparison clinics to be offered an HIV test.

**Table 2:** Modified Poisson regression of factors associated with HIV test outcomes among patients treated for STI, Botswana 2004

Independent variables (1)	Talked privately with provider about HIV test*	Offered HIV test*	Accepted HIV test*
	Relative risk (2) (95% CI* (3))	Relative risk (4) (95% CI* (5))	Relative risk (6) (95% CI* (7))
Training clinic			
No (n = 124)	Reference	Reference	Reference
Yes (n = 185)	**2.55** (**1.96 to 3.32**)	**2.92** (**2.19 to 3.90**)	0.88 (0.58 to 1.35)
Profession of healthcare provider			
Nurse (n = 223)	Reference	Reference	Reference
Nurse midwife (n = 77)	**1.17** (**1.04 to 1.32**)	**1.16** (**1.01 to 1.33**)	0.99 (0.68 to 1.44)
Medical officer (n = 9)	1.00 (0.68 to 1.46)	0.86 (0.48 to 1.54)	1.16 (0.45 to 3.03)
Visit type			
First visit (n = 229)	Reference	Reference	Reference
Follow-up visit (n = 66)	0.97 (0.84 to 1.11)	1.03 (0.88 to 1.20)	0.60 (0.36 to 1.01)
Patient gender			
Female (n = 215)	Reference	Rreference	Reference
Male (n = 91)	0.90 (0.74 to 1.11)	0.92 (0.72 to 1.17)	1.35 (0.77 to 2.38)
Syndromes			
Genital ulcer disease (n = 73)	Reference	Reference	Reference
VD/LAP (n = 171)	0.91 (0.76 to 1.08)	0.91 (0.73 to 1.14)	0.83 (0.50 to 1.40)
Urethral discharge (n = 36)	1.06 (0.80 to 1.41)	1.01 (0.75 to 1.35)	0.97 (0.57 to 1.64)
No symptoms (n = 29)	0.89 (0.71 to 1.13)	0.89 (0.68 to 1.16)	0.72 (0.34 to 1.49)
Visit observed by clinical specialist			
No (n = 211)	Reference	Reference	Reference
Yes (n = 98)	1.08 (0.98 to 1.20)	0.97 (0.87 to 1.10)	0.89 (0.60 to 1.32)
Understood right to refuse test			
No (n = 99)	Reference	Reference	Reference
Yes (n = 210)	**1.19** (**1.02 to 1.39**)	1.14 (0.96 to 1.36)	0.91 (0.62 to 1.34)
Sample size	305	304	193

STI, Sexually transmitted infection; VD/LAP, vaginal discharge or lower abdominal pain.

*Modified Poisson regressions adjust for health worker profession (nurse, nurse midwife or medical officer), follow-up visit, patient gender, syndromes, observation by a clinical specialist and before coming to the clinic whether or not the patient understood that s/he had the right to refuse an HIV test. †Results with a p value less than 0.05 are highlighted in bold.

The percentage of patients who refused an HIV test because they had already had one was similar in both samples; 29% for training and 28% for comparison clinics (p = 0.581). When these patients were omitted from the sample, the results reported above did not change substantially.

### Physical examination

Patients at training clinics were significantly more likely to report having a physical examination than at comparison clinics (98% versus 64%, respectively, p<0.001). Multivariate comparisons also showed significant differences between samples; patients at training clinics were 1.52 (95% CI 1.34 to 1.73) times more likely than at comparison clinics to have a physical examination.

### Patient satisfaction with quality of care

Patients at training clinics rated the quality of care more highly than at comparison clinics. After adjusting for differences between training and comparison clinics (columns 6 and 7 of table 3), the relative risk was significantly higher that patients at training than comparison clinics would strongly agree or agree with the following statements: (1) Did you feel the health worker gave you treatment for your problem?; (2) Did the health worker give you information about the nature of your problem?; (3) Did the health worker help you make a plan so that you could better prevent this problem in the future? and (4) On the whole, were you satisfied with the care you received for your problem today?

**Table 3:** Percentage of patients who strongly agreed or agreed with quality of care statements at clinics with the STI syndromic management training programme and comparison clinics, Botswana 2004

Statement (1)	Bivariate results				Multivariate results
	Training (%) (2)	Comparison (%) (3)	p Value (4)	Relative risk (2)/(3)	Adjusted relative risk* (6) (95% CI (7))
Did you believe that the information you shared about yourself with the health worker would be kept confidential?	92	87	0.120	1.06	1.04 (0.95 to 1.15)
Did the health worker give you enough opportunity to explain your problem?	98	99	0.652†	0.99	1.00 (0.98 to 1.02)
Did you feel comfortable asking the health worker questions about your problem?	95	88	0.020	1.08	1.07 (0.98 to 1.17)
Did you feel comfortable talking about your sexual behaviours with the health worker?	96	89	0.035	1.08	1.04 (0.95 to 1.13)
Did you feel comfortable sharing information about your sexual partner(s) with the health worker?	93	84	0.006	1.11	1.08 (0.96 to 1.20)
Do you believe the health worker accurately identified your problem?	92	86	0.049	1.07	1.06 (0.96 to 1.17)
Did you believe the health worker gave you treatment for your problem?	83	67	0.001	1.24	1.20 (1.02 to 1.40)
Did the health worker give you information about the nature of your problem?	90	72	<0.001	1.25	1.16 (1.01 to 1.34)
Did the health worker help you make a plan so that you could better prevent this problem in the future?	95	76	<0.001	1.25	1.21 (1.08 to 1.35)
On the whole, were you satisfied with the care you received for your problem today?	94	87	0.034	1.08	1.10 (1.02 to 1.19)
Sample size	185	124			

STI, Sexually transmitted infection.

*Modified Poisson regressions adjust for health worker profession (nurse versus nurse midwife), follow-up visit, syndromes and observation by a clinical specialist.

†Test statistic is from a Fisher’s exact test, because some of the cells in this analysis have an expected frequency of less than five.

### Patient education

Patients at training clinics were significantly more likely to report what they had learned about their HIV risk from the provider than at comparison clinics (65% versus 32%, respectively, p<0.001). Patients were classified as reporting no information from the provider when they said that they did not know or did not remember what the provider said, or that the provider did not discuss the topic. The multivariate comparisons showed that patients at training clinics were 1.72 (95% CI 1.22 to 2.40) times more likely than at comparison clinics to report receiving information about the risk of HIV.

## DISCUSSION

Botswana’s new training programme for the revised syndromic management protocols was associated with significant improvements in four outcomes: (1) routine HIV testing; (2) physical examination; (3) risk-reduction counselling and (4) patient education about HIV risk. The providers were significantly more likely to offer an HIV test and overall patients were significantly more likely to have an HIV test at training than at comparison clinics. The likelihood of accepting an HIV test among patients who were offered one was the same for both samples, so having an HIV test depended on whether or not a provider offered it.

This is among the first reports of routine HIV testing for STI patients in a resource-limited setting since June 2004, when the World Health Organisation and the Joint United Nations Programme on HIV/AIDS recommended routine HIV testing in STI clinics or other clinics that provide STI care.^22^ In Botswana, the difference in the percentage of patients who had an HIV test between training (33%) and comparison clinics (14%) was within the range reported in England by Day *et al*.^23^

Providers who attended the new STI training programme were significantly more likely to conduct a physical examination, help patients to make a plan to avoid acquiring STI in the future and discuss HIV risks with patients. Our findings add substantially to the relatively limited body of research demonstrating an effect of STI training in resource-limited settings on conducting a physical examination^1^ ^7^ and counselling STI patients on their HIV risk.^5^ ^7^ ^24^

This study is among the first to use patient reports on whether or not tasks were performed by providers. Although patient exit interviews are a well-known method for evaluating the quality of STI care,^25^ previous researchers have only used them to collect information on patient education,^7^ ^26^ ^27^ opinions on waiting time and satisfaction with care,^27^ condoms^7^ ^26^ and contact slips.^26^ Some researchers consider unannounced (or blinded) standardised patient encounters to be the “gold standard” for measuring the quality of clinical practice.^28^ Standardised patients can not be used, however, to evaluate some aspects of quality, such as physical examination and the diagnosis of an STI based on clinical symptoms.

The study had several limitations. First, the design was limited to a single time period after training providers at the training sites, so differences between districts could potentially have been confounded with the effects of the training programme. Health districts were, however, selected to minimise differences among clinics and patients and multivariate analyses adjusted for differences between the samples. Second, it is possible that patient visits at training clinics that were not observed were influenced by the recent presence of a clinical specialist. The clinical specialist, however, was not at training clinics on the days when observations were not performed. Third, the analysis may not have fully adjusted for unobserved differences among providers and clinics; random effects analysis of variance regressions would be necessary to adjust fully for these differences. Given the magnitude of the differences between patient reports at training and comparison clinics, however, it is unlikely that the additional analysis would alter the conclusions for the four main outcomes. Fourth, HIV testing results do not include whether or not patients learned their HIV test results. Fifth, the data were collected within two months of training and can not show whether or not the effects of training persisted over time. Future activities will include mentoring and supervision visits for trainees to reinforce the training programme and learn whether or not the results persisted. Finally, the percentage of patients who strongly agreed or agreed with statements about quality of care was high, which could accurately reflect patient experience or could reflect acquiescent response bias.^29^ ^30^ To the extent that bias existed, it would not be more likely to occur in training than comparison clinics.

Future studies on the outcomes of training programmes should have a more rigorous quasi-experimental design, with baseline and post-training data. In these studies, the provider or clinic should be the unit of analysis, with a sample of a relatively large number of providers or clinics and a small number of patients per provider or clinic. The analysis should account for clustering at the provider and clinic level.

In conclusion, a multifaceted training programme was associated with higher rates of HIV testing, physical examination, risk-reduction counselling and better HIV risk education.
